# Identification of an exported heat shock protein 70 in *Plasmodium falciparum*

**DOI:** 10.1051/parasite/2012002

**Published:** 2013-01-14

**Authors:** Manish Grover, Shweta Chaubey, Shatakshi Ranade, Utpal Tatu

**Affiliations:** Department of Biochemistry, Indian Institute of Science Bangalore 560012 Karnataka India

**Keywords:** *Plasmodium falciparum*, Chaperone, Hsp70, Export, Trafficking

## Abstract

Host cell remodelling is a hallmark of malaria pathogenesis. It involves protein folding, unfolding and trafficking events and thus participation of chaperones such as Hsp70s and Hsp40s is well speculated. Until recently, only Hsp40s were thought to be the sole representative of the parasite chaperones in the exportome. However, based on the re-annotated *Plasmodium falciparum* genome sequence, a putative candidate for exported Hsp70 has been reported, which otherwise was known to be a pseudogene. We raised a specific antiserum against a C-terminal peptide uniquely present in PfHsp70-*x*. Immunoblotting and immunofluorescence-based approaches in combination with sub-cellular fractionation by saponin and streptolysin-O have been taken to determine the expression and localization of PfHsp70-*x* in infected erythrocyte. The re-annotated sequence of PfHsp70-*x* reveals it to be a functional protein with an endoplasmic reticulum signal peptide. It gets maximally expressed at the schizont stage of intra-erythrocytic life cycle. Majority of the protein localizes to the parasitophorous vacuole and some of it gets exported to the erythrocyte compartment where it associates with Maurer’s clefts. The identification of an exported parasite Hsp70 chaperone presents us with the fact that the parasite has evolved customized chaperones which might be playing crucial roles in aspects of trafficking and host cell remodelling.

## Abstract (fr)

La réorganisation de la cellule hôte est une caractéristique de la pathogenèse de la malaria. Elle implique le repliement et le dépliement de protéines et des événements de trafic, et la participation de chaperons tels que Hsp70 et Hsp40 a donc été spéculée. Jusqu’à récemment, on pensait que les protéines Hsp40 étaient les seules représentantes des chaperons parasitaires dans l’exportome. Toutefois, sur la base d’une séquence ré-annotée du génome de *Plasmodium falciparum*, un candidat possible pour une Hsp70, qui autrement était connu comme pseudogène, a été signalé. Nous avons produit un antisérum spécifique contre un peptide C-terminal présent uniquement chez PfHsp70-x. Nous avons utilisé des approches basées sur l’immunomarquage et l’immunofluorescence en combinaison avec le fractionnement subcellulaire par la saponine et le streptolysine-o pour déterminer l’expression et la localisation de PfHsp70-x dans l’érythrocyte infecté. La séquence ré-annotée de PfHsp70-x s’est révélée être une protéine fonctionnelle avec un peptide de signal du réticulum endoplasmique. Elle est exprimée au maximum dans le stade schizonte du cycle intra-érythrocytaire. La majorité de la protéine se localise dans la vacuole parasitophore et une partie est exportée vers le compartiment érythrocytaire où elle s’associe avec les fissures de Maurer. L’identification d’un chaperon Hsp70 parasitaire exporté signifie que le parasite a développé des chaperons adaptés qui pourraient jouer un rôle crucial dans des aspects du trafic et de la réorganisation de la cellule hôte.

**Abbreviations**
Hsp: Heat shock protein
ER: Endoplasmic reticulum
PV: Parasitophorous vacuole
PEXEL: *Plasmodium* export element
Hpi: Hours post-infection
DIC: Differential interference contrast
KAHsp40: Knob associated heat shock protein 40
MAHRP1: Membrane associated histidine-rich protein 1
KAHRP: Knob associated histidine-rich protein
PfEMP: *Plasmodium falciparum* erythrocyte membrane protein

## Introduction

Intra-erythrocytic stage of the malaria parasite *Plasmodium falciparum* is rich in endomembrane system. In addition to the compartments of exocytic and endocytic pathways found in other eukaryotic cells, *P. falciparum* infected erythrocytes harbour unique organelles such as Maurer’s clefts and tubulovesicular network. Understanding trafficking to these locales presents an interesting challenge to parasite cell biologists.

Recent years have seen significant progress in our understanding of how parasite exports proteins beyond the parasitophorous vacuolar membrane (PVM) into the erythrocyte cytosol and on the erythrocyte membrane. Many exported proteins have been shown to have a pentapeptide motif (R_L_E/Q/D), called as PEXEL, in their N-terminal region which is necessary for their export beyond the PVM [7, 12]. A translocon, called as PTEX, has also been identified in the PVM through which PEXEL containing proteins get exported into the erythrocyte cytosol [3]. A general phenomenon observed across different biological systems is that proteins get transported through the translocon in an unfolded state. This suggests that fully folded proteins which get secreted from the ER into the PV get unfolded for translocation [6] and refold to acquire functional conformation in the erythrocyte cytosol. Molecular chaperones may play an important role in this process owing to their ability to fold, unfold and stabilize proteins. Many exported proteins also have multiple homo-repeats and prion-like domains in their sequence which make them susceptible to aggregation [15]. This further emphasizes the involvement of chaperones in the trafficking of exported proteins.

The parasite is well equipped with a large repertoire of chaperones. Nearly, 2% of its genome is dedicated for this purpose and 18 proteins among them are predicted to be exported [2]. Surprisingly, all these exported chaperones are DNAJ proteins which belong to the Hsp40 class of chaperones. Not much is known about the localization and functions of these exported Hsp40s in the infected erythrocyte. Recently, a report from our laboratory implicated an exported Hsp40, called KAHsp40 (PFB0090c/ PF3D7_0201800), in the process of knob biogenesis [1]. Previously, two other exported Hsp40s were shown to be present in cholesterol associated mobile structures, called as J-dots, in the erythrocyte cytosol [9]. Signal peptide containing chaperones are probably not unique to *Plasmodium* species as a related apicomplexan, namely *Theileria*, also shows presence of signal peptide containing DNAJ homologues in the genome data. Here too, these secreted Hsp40’s may participate in remodelling of host cell compartment.

Hsp40s usually function with an Hsp70 partner. There are six Hsp70 proteins present in *P. falciparum*, namely PfHsp70-1,2,3 and PfHsp70-*x*,*y*,*z* [14]. PfHsp70-1, 2 and 3 are the canonical cytosolic (PF08_0054/PF3D7_0818900), ER (PFI0875w/PF3D7_0917900) and mitochondrial Hsp70 (PF11_0351/ PF3D7_1134000) respectively. PfHsp70-*y* (MAL13P1.540/PF3D7_1344200) and PfHsp70-*z* (PF07_0033/ PF3D7_0708800) are the nucleotide exchange factors present in ER and cytosol respectively. The sixth member of this class is PfHsp70-*x* (MAL7P1.228/PF3D7_0831700). It is 73% identical to PfHsp70-1 and also contains an EEVN motif at the C-terminus [14]. Until recently, this protein had been annotated as a pseudogene since it had an in-frame stop codon in the N-terminal region. None of these PfHsp70s possess the PEXEL motif required for export into the erythrocyte compartment.

The re-annotation of *P. falciparum* genome sequence has identified a single base change in PfHsp70-*x* sequence which resulted in the loss of the in-frame stop codon. According to the re-annotated sequence, the entire ORF codes for a functional protein and it also possesses a hydrophobic sequence at the N-terminus which could serve as a potential ER signal peptide. In this study, we have examined the expression, localization and sub-cellular distribution of PfHsp70-*x*. Using a peptide epitope uniquely present in PfHsp70-*x*, specific antiserum was generated against this protein. As opposed to the transcriptomic profile of PfHsp70-*x* where mRNA expression peaks in the ring stage of parasite life cycle, we find that protein expression is maximal in the schizont stage. By immunoblotting approach in combination with sub-cellular fractionation using saponin and streptolysin-o, we report that majority of this protein is present in the PV apart from within the parasite and a small fraction (~30%) gets exported to the erythrocyte compartment. As revealed by indirect immunofluorescence approach, the exported population forms punctate spots in the erythrocyte periphery which partially overlap with Maurer’s clefts. In all, our results highlight the first ever parasite encoded Hsp70 to be found exported in the erythrocyte compartment. The results provide an important implication on protein folding, unfolding and trafficking events in this strategic location for intra-erythrocytic growth and development.

## Materials and methods

### Continuous culturing of parasites

*Plasmodium falciparum* 3D7 strain was cultured in human O+ve erythrocytes at 5% haematocrit in RPMI 1640 medium (Sigma Aldrich) supplemented with 200 *μ*M hypoxanthine, 0.2% (w/v) sodium bicarbonate, 0.2% (w/v) glucose and 0.5% (w/v) albumax II (Invitrogen). Parasites were synchronized by 5% sorbitol treatment as described previously [10].

### Antibodies

PfHsp70-*x* polyclonal antiserum was raised in mice and rabbit against a C-terminal peptide “QKAEATNLRGRNSENKEA” from PfHsp70-*x* sequence. *α*-PTEX150 antiserum was used as described previously [1]. *α*-MAHRP1, *a*-KAHRP1 and *a*-PfBip antibodies were obtained from Prof. Hans Peter Beck, Prof. Diane Taylor and MR4 respectively. Animal handling was done adhering to the institution’s guidelines for animal husbandry at the Indian Institute of Science.

### Parasite fractionation

Parasite infected erythrocytes were separated from the uninfected cells by using a 60% Percoll gradient. These cells were divided into two parts. One part was treated with saponin (0.07% in pH 7.4 PBS) and incubated on ice for 10 min and then centrifuged at 2,500 g for 10 min. The other part was subjected to treatment with 200 U of activated Streptolysin O (SLO; Sigma Aldrich) at 37 °C for 15 min and then centrifuged at 1,500 g for 10 min. The fractions were solubilized in Laemmli buffer and analysed by 10% SDS-PAGE followed by immunoblotting. All the lysis procedures were carried out in the presence of protease inhibitor cocktail (Roche).

### Two-dimensional electrophoresis

It was performed as described previously by O’Farrell [13]. Briefly, infected erythrocyte lysate was acetone precipitated and the protein pellet was dissolved in 2D lysis buffer (9.5 M urea, 2% NP-40, 2% ampholines and 5% DTT). Isoelectric focusing (IEF) gel was polymerized in tube (7 cm × 1.5 mm) followed by pre-focusing of this IEF tube gel at 250 V for 30 min. Proteins were then loaded onto tube gel and resolved at 500 V for 4 h. Tube gels were equilibrated in a buffer containing 125 mM Tris-HCl, pH 6.8, 2% SDS, 4.9 mM DTT and 10% glycerol for 10 min. For the second dimension, these tube gels were laid horizontally on the top of vertical SDS-PAGE.

### Indirect immunofluorescence analysis

It was performed as previously described by Tonkin *et al*. [16]. Briefly, cells were washed once in PBS then fixed with 4% EM grade paraformaldehyde (ProSciTech) and 0.0075% EM grade glutaraldehyde (ProSciTech) in PBS for 30 min. Fixed cells were washed once in PBS and then permeabilized with 0.1% Triton X-100/PBS for 2 min. Cells were then blocked in 3% BSA/PBS for one hour. Anti-PfHsp70-*x*, anti-MAHRP1 or anti-KAHRP1 antibody (1:50 dilution) was added and allowed to bind for a minimum of 1 h in 3% BSA/PBS. Cells were washed three times in PBS for 10 min each to remove excess primary antibody. FITC-conjugated goat anti-rabbit and TRITC-conjugated goat anti-mice were added at 1:300 dilution (in 3% BSA/PBS) and allowed to bind for an hour. Cells were washed three times in PBS and mounted in 70% glycerol with 2% DABCO. The coverslips were then inverted onto a glass microscope slide, mounted and sealed.

## Results

### Re-annotation of PfHsp70-*x* sequence

**Figure 1.**
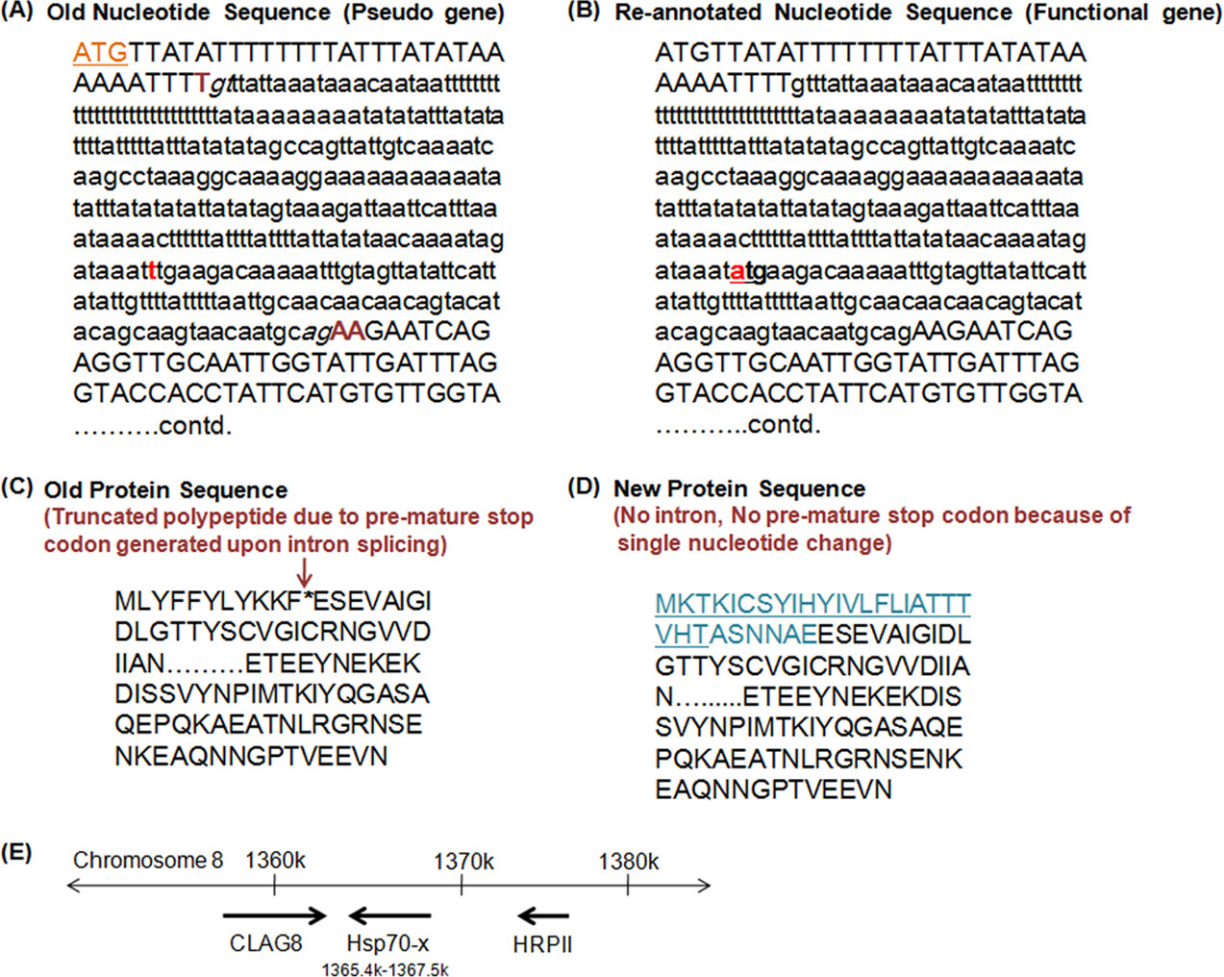
*Re-annotation of PfHsp70-x sequence*: (A) Original nucleotide sequence – intron is shown in lower case with splice sites in italics. ATG represents the start site, TAA is the in-frame stop codon created upon splicing and **t** is the erroneously designated SNP in the intron. (B) New nucleotide sequence – **atg** represents the alternate start site annotated following **t** > **a** change in the nucleotide sequence. Now the gene lacks intron and encodes the entire ORF. Sequence in lower case before **atg** is now a part of 5′ UTR whereas the sequence after ***atg*** is part of the ORF. (C) Original protein sequence – * represents the in-frame stop codon which resulted in a truncated polypeptide. (D) New protein sequence – the sequence in blue is the new N-terminal sequence and underlined region represents the putative signal peptide. (E) Genomic location of PfHsp70-*x*.

**Figure 2.**
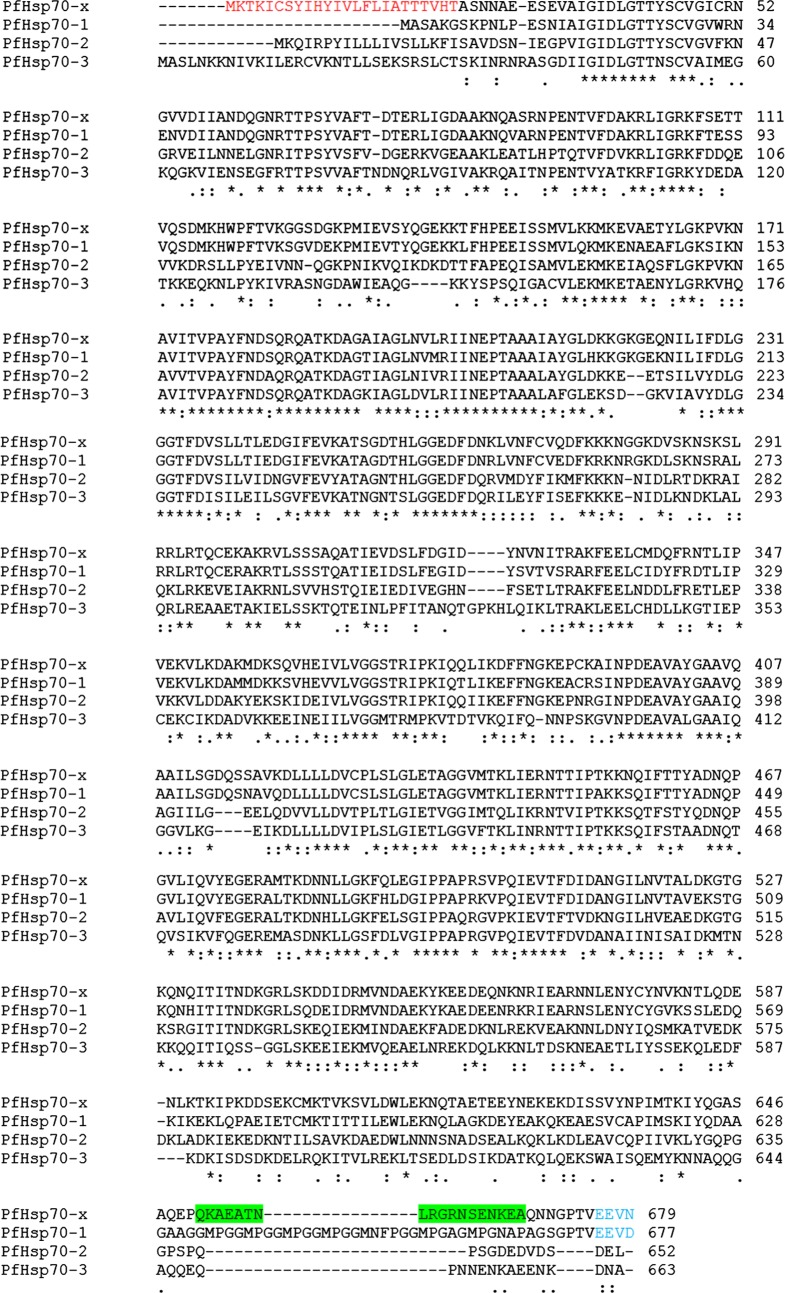
*Sequence alignment of Hsp70s in P. falciparum*: PfHsp70-*x* is aligned with PfHsp70-1 (cytosolic), PfHsp70-2 (ER) and PfHsp70-3 (mitochondrial) using ClustalW. Red: putative signal peptide, blue: EEVD/EEVN motif, green: peptide used to raise specific antiserum against PfHsp70-*x* in both mice and rabbit.

The original annotation of PfHsp70-*x* in PlasmoDB 8.2 described it as a pseudogene. It was shown to be encoded by two exons and upon splicing of the intron an in-frame stop codon (TAA) was generated towards the 5′ end of the gene (Figure 1A and B). In the predicted intron sequence of PfHsp70-*x* a SNP was present, which was T in 3D7 and A in other strains of *P. falciparum* (combined SNP.MAL7.1058 in PlasmoDB). It was recently found out that this SNP was erroneously designated in the original sequencing data and 3D7 also has A at this position like all other *P. falciparum* strains. As a result, an alternate start site (ATG) was generated (Figure 1A and B). The re-annotated coding sequence of PfHsp70-*x* now begins within the predicted intron sequence and encodes the entire ORF (Figure 1C and D). The new PfHsp70-*x* sequence lacks intron and encodes for a full-length Hsp70 protein showing overall sequence conservation with other PfHsp70s (Figure 2). Of the 90 nucleotides added at the 5′ end of the gene from the original intron sequence, the first 72 nucleotides have been predicted to code for an N-terminal signal peptide (Figure 1D). Figure 2 shows sequence alignment of PfHsp70-*x* with other Hsp70s of *P. falciparum*. Since PfHsp70-*x* is highly similar to the cytosolic Hsp70 (PfHsp70-1), it might have evolved as a result of gene duplication and later acquired additional features. Since this gene is uniquely present in *P. falciparum*, it is likely to be involved in aspects of host-cell remodelling, a special feature of *P. falciparum* pathogenesis.

PfHsp70-*x* lies in the sub-telomeric region on chromosome 8 adjacent to hrpii gene and clag8 (Figure 1E). This region is highly vulnerable to chromosomal breakage in both *in vitro P. falciparum* cultures and in parasites isolated from patients [5]. However, loss of PfHsp70-*x* gene has not been reported from any of the parasite lines studied so far [5]. This indicates that PfHsp70-*x* might be playing an essential role in the parasite life cycle.

### Specificity of PfHsp70-*x* antiserum

**Figure 3.**
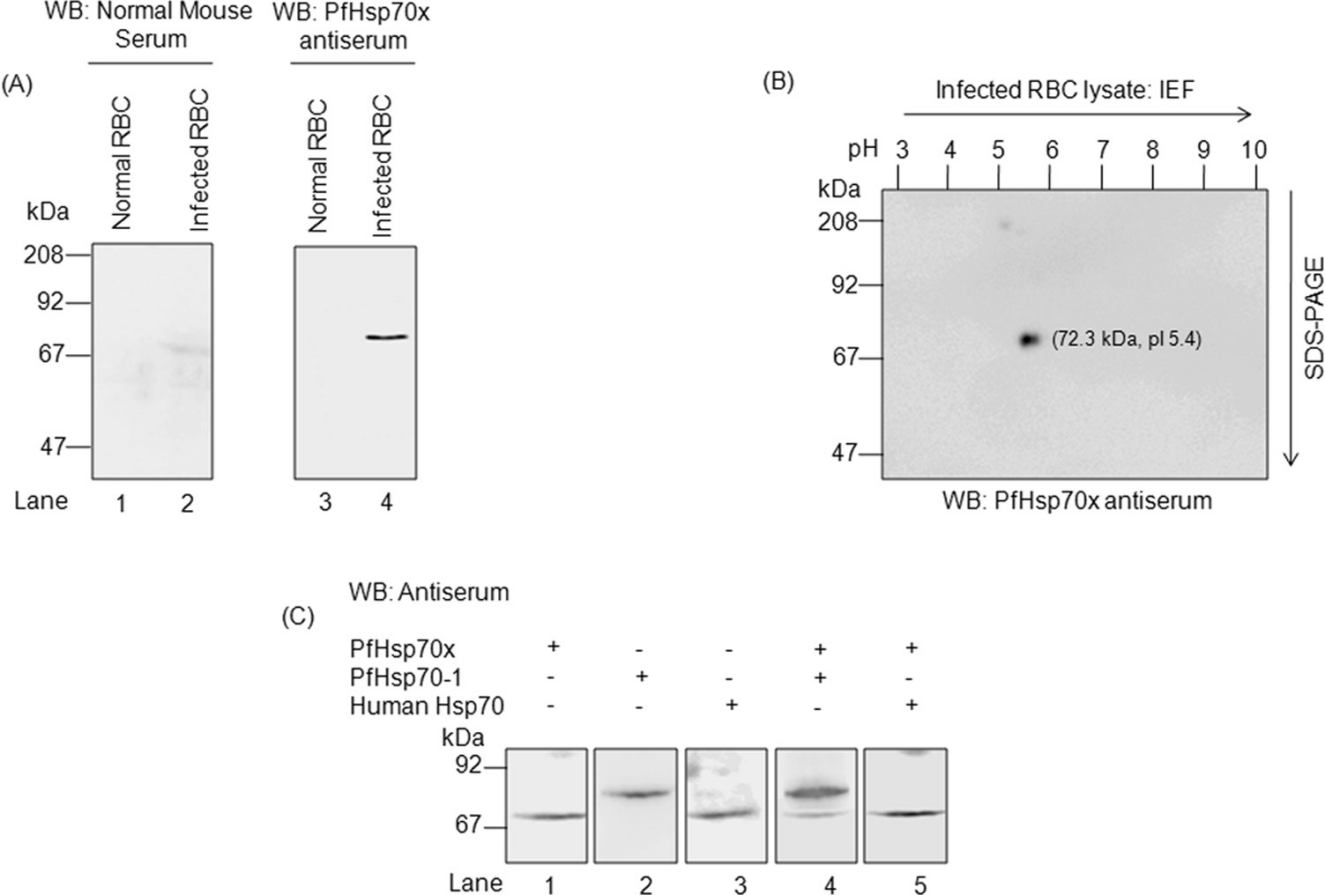
*Specificity of PfHsp70-x antiserum*: (A) Total lysates from normal and infected erythrocytes analysed by immunoblotting with normal mouse serum (left) and PfHsp70-*x* antiserum (right). (B) Two-dimensional electrophoresis performed using lysate from parasite infected erythrocytes and probed with PfHsp70-*x* antiserum. (C) Lysates from infected erythrocytes analysed by immunoblotting with other host and parasite heat shock protein antisera. Lane 1: probed with PfHsp70-*x* antiserum, Lane 2: probed with PfHsp70-1 antiserum, Lane 3: probed with Host Hsp70 antiserum, Lane 4: probed with both PfHsp70-*x* and PfHsp70-1 antiserum and Lane 5: probed with both PfHsp70-*x* and Host Hsp70 antiserum. The blots indicate that PfHsp70-*x* antiserum is specific and does not cross-react with any other host or parasite chaperone.

The sequence alignment in Figure 2 shows an underlined region towards the C-terminus of PfHsp70-*x* which is uniquely present in this protein. This peptide sequence was used to raise a specific antibody against PfHsp70-*x* in mice and rabbit. To test the specificity of the raised antiserum, total lysates from normal and Percoll-purified infected erythrocytes were solubilized in Laemmli buffer and analysed by immunoblotting. As shown in Figure 3A, when normal and infected samples were probed with PfHsp70-*x* antiserum, only the infected cells showed a band with a molecular mass of about 72 kDa corresponding to PfHsp70-*x*. To further confirm the identity of the recognized band, two-dimensional electrophoresis was performed using infected erythrocyte lysate followed by immunoblotting with PfHsp70-*x* antiserum. As we can see in Figure 3B, a single spot which corresponds to the molecular weight and pI of PfHsp70-*x* protein (MW and pI of mature form of PfHsp70-*x* is 72.3 kDa, 5.4) was obtained. In order to rule out that PfHsp70-*x* antiserum does not cross-react with any other host or parasite Hsp70s, infected erythrocyte lysate was analysed by immunoblotting with mixture of antibodies. Figure 3C shows that when antisera against PfHsp70-1 and PfHsp70-*x* were used together, two bands were obtained. The one with higher MW corresponded to PfHsp70-1 (75 kDa) whereas the lower MW band represented PfHsp70-*x* (72.3 kDa). The difference between the molecular weights of PfHsp70-*x* and host Hsp70 is just 1 kDa and their pIs are also identical. As a result, it is difficult to observe a mobility difference between the two proteins on SDS-PAGE or 2D-electrophoresis. Since, PfHsp70-*x* antiserum does not recognize any band in normal erythrocytes, it confirms that PfHsp70-*x* antiserum does not recognize the Hsp70 of host origin. Hence, the antiserum raised against PfHsp70-*x* is a specific one.

### PfHsp70-*x* protein is maximally expressed in the schizont stage

**Figure 4.**
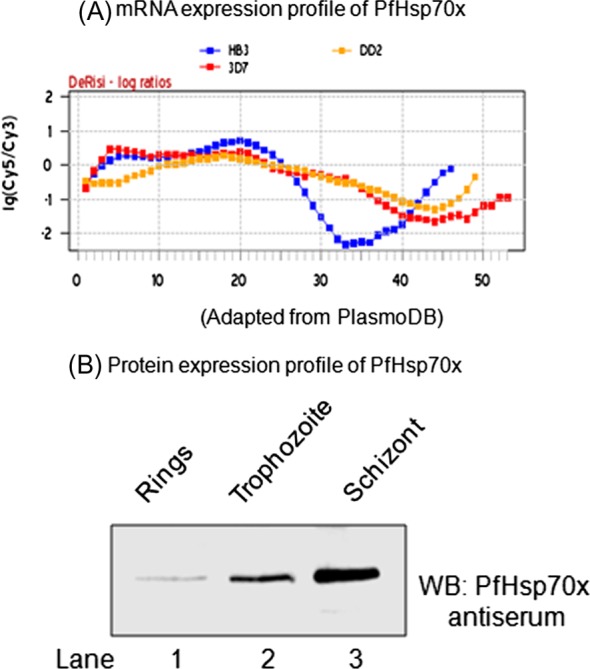
*Expression profile of PfHsp70-x*: (A) mRNA expression profile of PfHsp70-*x* during the 48 h asexual cycle from three different strains of *P. falciparum* (red: 3D7, blue: HB3 and yellow: DD2), adapted from PlasmoDB. (B) Western blot analysis of total parasite lysate at different stages of *P. falciparum* asexual cycle. Ring stage (2–8 hpi), Trophozoite stage (24–30 hpi) and Schizont stage (36–48 hpi); hpi: hours post-infection.

The previous transcription analysis has revealed high PfHsp70-*x* mRNA levels in the early stage of the parasite life cycle [11]. The mRNA levels peak during ring stage (2–16 h) and gradually decline attaining the lowest level around 34–38 h, i.e., during early schizont stage (Figure 4A). To examine whether the transcriptomic profile of PfHsp70-*x* correlates with its proteomic expression, we performed immunoblot analysis for PfHsp70-*x* at different stages of the parasite life cycle. Towards this, parasites were isolated from a tightly synchronized culture at ring (2–12 hpi), trophozoite (24–30 hpi) and schizont (36–48 hpi) stages (hpi: hours post-infection). The parasite lysate was quantified and equal protein from each stage was loaded onto a SDS-PAGE gel and subjected to immunoblotting with PfHsp70-*x* antiserum. As we can see in Figure 4B, PfHsp70-*x* protein expression progressively increases during the asexual cycle attaining maximum level in schizont stage. This indicates that proteomic profile of PfHsp70-*x* does not follow the same pattern as mRNA expression profile.

### PfHsp70-*x* localizes to the PV as well as erythrocyte cytosol

**Figure 5.**
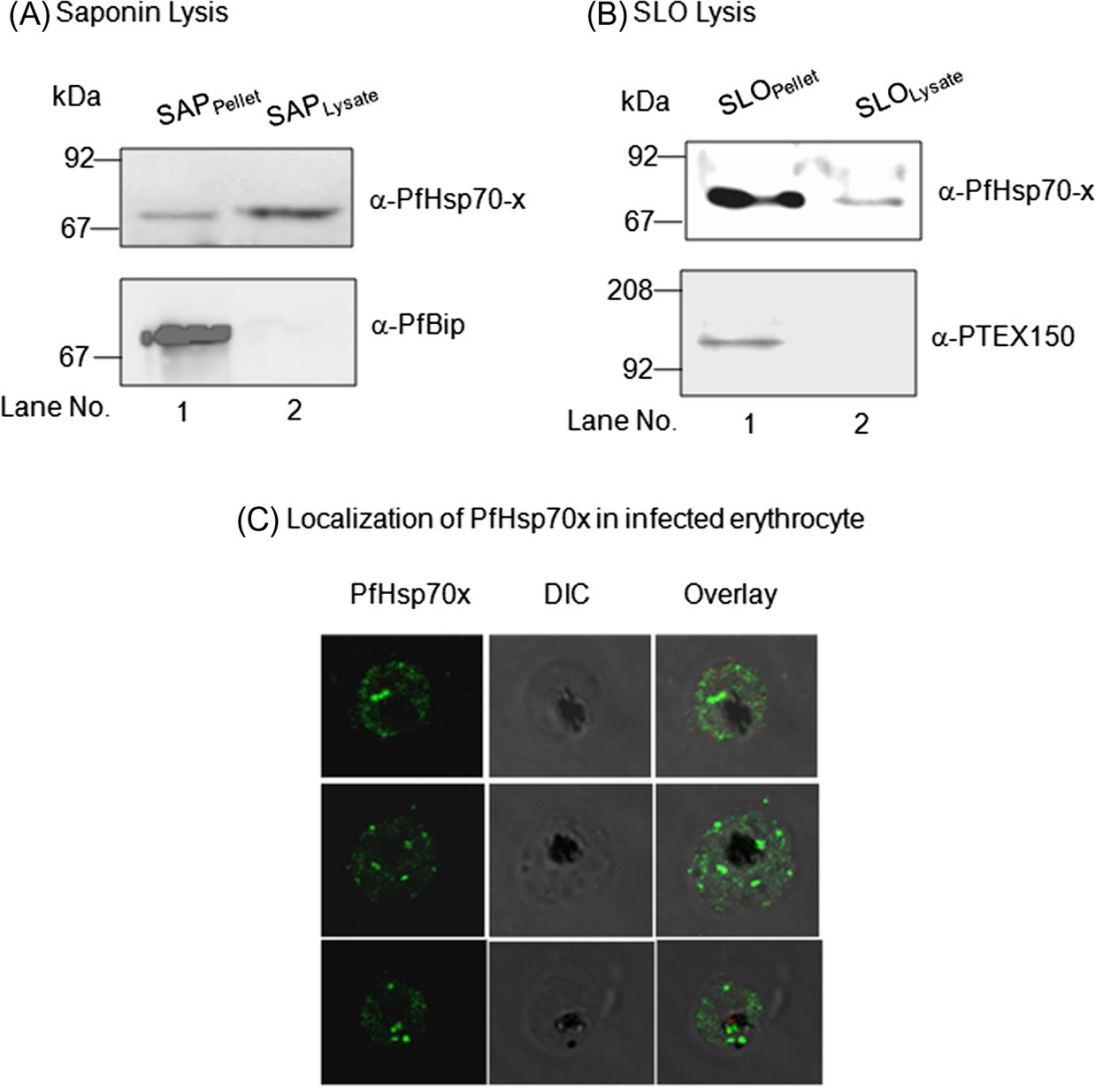
*Sub-cellular localization of PfHsp70-x in infected erythrocyte*: Infected erythrocytes were lysed with saponin (A) and SLO (B) and the fractions obtained were analysed by immunoblotting with PfHsp70-*x* antiserum (top). To ensure that compartmental integrity was maintained during saponin and SLO-based fractionation, the fractions were immunoblotted with PfBip and PTEX150 respectively (below). PfHsp70-*x* gets exported outside the parasite with major population being present in the PV and some in the erythrocyte compartment. (C) IFA analysis of infected erythrocytes with PfHsp70-*x* antiserum reveals its presence throughout the infected erythrocyte.

The presence of an N-terminal signal peptide in PfHsp70-*x* sequence suggests that it would be targeted to the ER and secreted into the parasitophorous vacuole (PV). Since it lacks the canonical PEXEL motif required for protein export to erythrocyte, it is less likely that it would be exported to the erythrocyte cytosol. However, there are evidences of PEXEL-independent export for many parasite proteins as well. Therefore, we decided to examine the sub-cellular distribution of PfHsp70-*x* in the infected erythrocyte. Towards this, saponin and SLO-based fractionation of infected erythrocytes was performed and the fractions were analysed by immunoblotting with PfHsp70-*x* antiserum. Saponin will cause lysis of both erythrocyte membrane and PVM thereby separating out the parasites whereas SLO will specifically lyse only the erythrocyte membrane leaving the PVM intact along with the parasites. As we can see in Figure 5A and B, PfHsp70-*x* was present in both saponin pellet (SP) and lysate (SL) fractions as well as SLO pellet and SLO lysate. This suggests that PfHsp70-*x* gets exported to the erythrocyte cytosol even though it lacks the canonical PEXEL motif required for export. Immunoblot for PfBiP and PTEX150 was performed to ensure that compartmental integrity was maintained during saponin and SLO-based fractionation (Figure 5A and B).

The localization of PfHsp70-*x* in the infected erythrocyte was further confirmed by indirect immunofluorescence analysis (IFA). PfHsp70-*x* was stained green with FITC-conjugated secondary antibody. As we can see in Figure 5C, the signal for PfHsp70-*x* can be seen as punctate spots in the erythrocyte cytosol and around the PVM apart from signal within the parasite. More amount of signal was observed on the inner side of PVM, suggesting that PfHsp70-*x* gets exported partially, further supporting the immunoblot result described above.

A recently published independent study [8] has also reported similar findings and has elegantly described the export of PfHsp70-*x* in the erythrocyte compartment.

### PfHsp70-*x* partially associates with Maurer’s clefts

**Figure 6.**
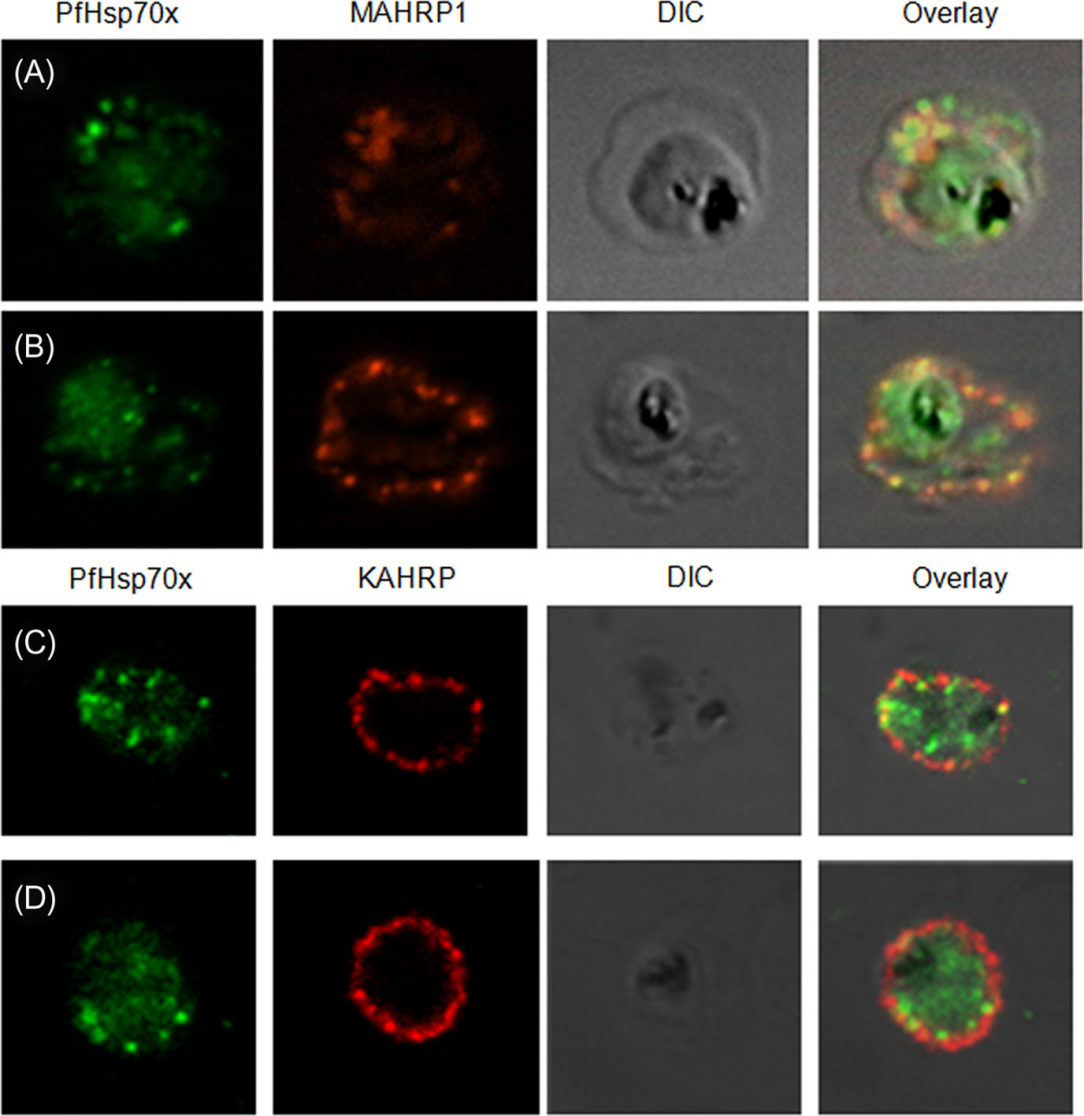
*PfHsp70-x partially associates with Maurer’s clefts but not with knobs*: (A–B) Panel I: the signal for PfHsp70-*x* (green) was obtained in the parasite compartment along with few punctate spots in the erythrocyte compartment. Panel II: MAHRP1 (red) stained discrete foci representative of Maurer’s clefts in the erythrocyte periphery. Panel III: the DIC image of the infected erythrocyte. Panel IV: merged image overlaid with DIC image reveals that MAHRP1 and PfHsp70-*x* partially co-localize in the erythrocyte compartment, suggesting that PfHsp70-*x* possibly associates with Maurer’s clefts. (C-D) Panel I: the signal for PfHsp70-*x* (green) was obtained in the parasite compartment along with few punctate spots in the erythrocyte compartment. Panel II: KAHRP (red), being a constituent of knobs, stained the entire erythrocyte membrane. Panel III shows the DIC image of the infected erythrocyte. Panel IV: no co-localization is observed between KAHRP and PfHsp70-*x*, suggesting that PfHsp70-*x* does not associate with knobs on the infected erythrocyte membrane.

Maurer’s clefts are the pathogen-induced major secretory organelles present in the erythrocyte compartment. These clefts have been implicated in the sorting of several membrane as well as soluble exported proteins in the parasite infected erythrocyte. Having established that PfHsp70-*x* gets exported beyond the confines of the parasite boundary, we aimed to examine whether the protein associates with Maurer’s clefts. Therefore, we carried out double IFA for PfHsp70-*x* with MAHRP1, a well-characterized Maurer’s cleft marker. PfHsp70-*x* was detected with rabbit *α*-PfHsp70-*x* antiserum followed by FITC-conjugated secondary antibody (Figure 6A and B; first panel), MAHRP1 was detected with mouse α-MAHRP1 followed by TRITC-conjugated secondary antibody (Figure 6A and B; second panel) and the merged images were overlaid with DIC image (Figure 6A and B; third panel) in order to examine co-localization between the two proteins (Figure 6A and B; fourth panel). Signal for PfHsp70-*x* was found in the parasite as well as the erythrocyte compartment, majorly at the periphery. MAHRP1 formed punctate foci in the erythrocyte cytosol especially underneath the erythrocyte membrane showing partial co-localization with PfHsp70-*x*.

We further wanted to examine the association of PfHsp70-*x* with the major virulence determinant of the malaria parasite, i.e., knobs. Knobs, which are known to be the assemblage of the parasite exported proteins such as PfEMP1, KAHRP, PfEMP2, PfEMP3, provide the property of cytoadherence to the parasitized erythrocytes. Using KAHRP as a marker for the knobs, we carried out the indirect IFA study to determine the association of PfHsp70-*x* with the knobs. PfHsp70-*x* was detected with rabbit α-PfHsp70-*x* antiserum followed by FITC-conjugated secondary antibody (Figure 6C and D; first panel), KAHRP was detected with mouse α-KAHRP1 followed by TRITC-conjugated secondary antibody (Figure 6C and D; second panel) and the merged images were overlaid with DIC image (Figure 6C and D; third panel) in order to examine co-localization between the two proteins (Figure 6C and D; fourth panel). Signal for PfHsp70-*x* was found in the parasite as well as the erythrocyte compartment, majorly at the periphery. KAHRP formed punctate foci on the erythrocyte membrane. In spite of the proteins being in close proximity to each other, no convincing co-localization could be detected between them.

Therefore, PfHsp70-*x* partially associates with the Maurer’s cleft but does not show any physical overlap with the knobs.

## Discussion

The re-annotation of *P. falciparum* genome has provided details about an important missing link in the field of chaperone biology of the malaria parasite. Identification of the single base error in the original annotation of PfHsp70-*x* sequence has given some clue for the long-standing question of lack of an exported Hsp70 in *P. falciparum*. The new sequence of PfHsp70-*x* contains an ER signal peptide and thus satisfies the requirement to enter the secretory pathway. Intrigued by this observation and its unique presence only in *P. falciparum*, we carried out characterization of its expression and localization and also examined overlap with other compartments in the infected erythrocyte.

Based on the sequence alignment of PfHsp70s, a unique region present in the C-terminus of PfHsp70-*x* was identified. This peptide sequence was used to raise a specific antiserum against PfHsp70-*x* in both mice and rabbit. The PfHsp70-*x* antiserum did not show cross-reactivity to either PfHsp70-1 or human Hsp70. This also confirmed that antiserum recognized only the mature form of PfHsp70-*x* as molecular weight of PfHsp70-1 is same as the molecular weight of full-length PfHsp70-*x* with the signal peptide. The mRNA expression of PfHsp70-*x* is highest during the early stages of the parasite life cycle, whereas the protein expression was found to be maximal in the schizont stage. It is not surprising as it has been previously reported that there is a delay in mRNA expression and protein accumulation in *P. falciparum* [11].

Sub-cellular fractionation of infected erythrocytes using saponin and SLO revealed that PfHsp70-*x* is present in the PV as well as the erythrocyte cytosol. IFA further supported this finding as PfHsp70-*x* showed punctate distribution in the erythrocyte cytosol and around the PV apart from signal within the parasite. However, only a small fraction of PfHsp70-*x* was exported beyond the PVM into the erythrocyte cytosol, suggesting its potential involvement in processes on either side of PVM. A major population of the exported PfHsp70-*x* showed overlap with MAHRP1, a Maurer’s cleft marker, in the erythrocyte periphery. However, no co-localization was observed with KAHRP, the marker for cytoadherent knobs on the erythrocyte membrane. This suggested that PfHsp70-*x* associates with Maurer’s clefts and might be involved in protein sorting and trafficking activities.

Since, PfHsp70-*x* lacks the canonical PEXEL motif, it may follow some unusual pathway for its export to the erythrocyte cytosol. Among the 30 amino acids uniquely present at the N-terminus of PfHsp70-*x*, the first 24 represent the signal peptide. Therefore, the following hexameric sequence ASNNAE, before the beginning of the ATPase domain, could serve as the putative signal for export into the erythrocyte. This sequence is somewhat similar in terms of amino acid residues to the putative export sequence (*NS*IK*ENANS*K) for REX1, another exported protein lacking the PEXEL motif [4].

Regardless of the specific mechanisms required for PfHsp70-*x* export to the erythrocyte compartment, the presence of a bona fide parasitic Hsp70 member in the erythrocyte opens new possibilities to understand and explain protein homeostasis including folding, unfolding and trafficking events in the infected erythrocyte.

## References

[1] Acharya P, Chaubey S, Grover M, Tatu U. 2012 An exported heat shock protein associates with pathogenesis-related knobs in *Plasmodium falciparum* infected erythrocytes. PLoS One, 7, e44605 22970262 10.1371/journal.pone.0044605 PMC3436795

[2] Botha M, Pesce ER, Blatch G. 2007 The Hsp40 proteins of *Plasmodium falciparum* and other apicomplexa: regulating chaperone power in the parasite and the host. International Journal of Biochemistry and Cell Biology, 39, 1783–1803 10.1016/j.biocel.2007.02.011 17428722

[3] de Koning-Ward TF, Gilson PR, Boddey JA, Rug M, Smith BJ, Papenfuss AT, Sanders PRLundie RJ, Maier AG, Cowman AF, Crabb BS. 2009 A newly discovered protein export machine in malaria parasites. Nature, 459, 945–949 19536257 10.1038/nature08104 PMC2725363

[4] Dixon MW, Hawthorne PL, Spielmann T, Anderson KL, Trenholme KR, Gardiner DL. 2008 Targeting of the ring exported protein 1 to the Maurer’s clefts is mediated by a twophase process. Traffic, 9, 1316–1326 18489703 10.1111/j.1600-0854.2008.00768.x

[5] Gamboa D, Ho M-F, Bendezu J, Torres K, Chiodini PL, Barnwell JW, Incardona S, Perkins M, Bell D, McCarthy J, Cheng Q. 2010 A large proportion of *P. falciparum* isolates in the Amazon region of Peru lack *pfhrp2* and *pfhrp3:* implications for malaria rapid diagnostic tests. PLoS One, 5, e8091 20111602 10.1371/journal.pone.0008091 PMC2810332

[6] Gehde N, Hinrichs C, Montilla I, Charpian S, Lingelbach K, Przyborski JM. 2009 Protein unfolding is an essential requirement for transport across the parasitophorous vacuolar membrane of *Plasmodium falciparum*, Molecular Microbiology, 71, 613–628 19040635 10.1111/j.1365-2958.2008.06552.x

[7] Hiller NL, Bhattacharjee S, van Ooij C, Liolios K, Harrison T, Lopez-Estrano C, Haldar K. 2004 A host-targeting signal in virulence proteins reveals a secretome in malarial infection. Science, 306, 1934–1937 15591203 10.1126/science.1102737

[8] Külzer S, Charnaud S, Dagan T, Riedel J, Mandal P, Pesce ER, Blatch GL, Crabb BS, Gilson PR, Przyborski JM. 2012 *Plasmodium falciparum*-encoded exported hsp70/hsp40 chaperone/co-chaperone complexes within the host erythrocyte. Cellular Microbiology, 14, 1784–1795 22925632 10.1111/j.1462-5822.2012.01840.x

[9] Kulzer S, Rug M, Brinkmann K, Cannon P, Cowman A, Lingelbach K, Blatch GL, Maier AG, Przyborski JM. 2010 Parasite-encoded Hsp40 proteins define novel mobile structures in the cytosol of the *P. falciparum*-infected erythrocyte. Cellular Microbiology, 12, 1398–1420 20482550 10.1111/j.1462-5822.2010.01477.x

[10] Lambros C, Vanderberg JP. 1979 Synchronization of *Plasmodium falciparum* erythrocytic stages in culture. Journal of Parasitology, 65, 418–420 383936

[11] Le Roch KG, Johnson JR, Florens L, Zhou Y, Santrosyan A, Grainger M, Yan SF, Williamson KC, Holder AA, Carucci DJ, Yates JR 3rd, Winzeler EA. 2004 Global analysis of transcript and protein levels across the *Plasmodium falciparum* life cycle. Genome Research, 14, 2308–2318 15520293 10.1101/gr.2523904 PMC525690

[12] Marti M, Good RT, Rug M, Knuepfer E, Cowman AF. 2004 Targeting malaria virulence and remodelling proteins to the host erythrocyte. Science, 306, 1930–1933 15591202 10.1126/science.1102452

[13] O’Farrell PH. 1975 High resolution two-dimensional electrophoresis of proteins. Journal of Biological Chemistry, 250, 4007–4021 236308 PMC2874754

[14] Shonhai A, Boshoff A, Blatch GL. 2007 The structural and functional diversity of Hsp70 proteins from *Plasmodium falciparum*, Protein Science, 16, 1803–1818 17766381 10.1110/ps.072918107 PMC2206976

[15] Singh GP, Chandra BR, Bhattacharya A, Akhouri RR, Singh SK, Sharma A. 2004 Hyper-expansion of asparagines correlates with an abundance of proteins with prion-like domains in *Plasmodium falciparum*, Molecular and Biochemical Parasitology, 137, 307–319 15383301 10.1016/j.molbiopara.2004.05.016

[16] Tonkin CJ, van Dooren GG, Spurck TP, Struck NS, Good RT, Handman E, Cowman AF, McFadden GI. 2004 Localization of organellar proteins in *Plasmodium falciparum* using a novel set of transfection vectors and a new immunofluorescence fixation method. Molecular and Biochemical Parasitology, 137, 13–21 15279947 10.1016/j.molbiopara.2004.05.009

